# Magnetic, Electronic, and Optical Studies of Gd-Doped WO_3_: A First Principle Study

**DOI:** 10.3390/molecules27206976

**Published:** 2022-10-17

**Authors:** Ali Bahadur, Tehseen Ali Anjum, Mah Roosh, Shahid Iqbal, Hamad Alrbyawi, Muhammad Abdul Qayyum, Zaheer Ahmad, Murefah Mana Al-Anazy, Eslam B. Elkaeed, Rami Adel Pashameah, Eman Alzahrani, Abd-ElAziem Farouk

**Affiliations:** 1 Department of Chemistry, College of Science and Technology, Wenzhou-Kean University, Wenzhou 325060, China; 2 Nanomagnetism Laboratory, Department of Physics, COMSATS University Islamabad, Islamabad 45550, Pakistan; 3 Department of Chemistry, School of Natural Sciences (SNS), National University of Sciences and Technology (NUST), H-12, Islamabad 46000, Pakistan; 4 Pharmaceutics and Pharmaceutical Technology Department, College of Pharmacy, Taibah University, Medina 42353, Saudi Arabia; 5 Department of Chemistry, Division of Science & Technology, University of Education, Lahore 54770, Pakistan; 6 Department of Chemistry, University of Wah, Quaid Avenue, Wah Cantt 47040, Pakistan; 7 Department of Chemistry, College of Science, Princess Nourah bint Abdulrahman University, P.O. Box 84428, Riyadh 11671, Saudi Arabia; 8 Department of Pharmaceutical Sciences, College of Pharmacy, AlMaarefa University, Riyadh 13713, Saudi Arabia; 9 Department of Chemistry, Faculty of Applied Science, Umm Al-Qura University, Makkah 24230, Saudi Arabia; 10 Department of Chemistry, College of Science, Taif University, P.O. Box 11099, Taif 21944, Saudi Arabia; 11 Department of Biotechnology College of Science, Taif University, P.O. Box 11099, Taif 21944, Saudi Arabia

**Keywords:** Gd-doped, WO_3_, first-principle study, antiferromagnetic, bandgap tuning

## Abstract

Tungsten trioxide (WO_3_) is mainly studied as an electrochromic material and received attention due to N-type oxide-based semiconductors. The magnetic, structural, and optical behavior of pristine WO_3_ and gadolinium (Gd)-doped WO_3_ are being investigated using density functional theory. For exchange-correlation potential energy, generalized gradient approximation (GGA+U) is used in our calculations, where U is the Hubbard potential. The estimated bandgap of pure WO_3_ is 2.5 eV. After the doping of Gd, some states cross the Fermi level, and WO_3_ acts as a degenerate semiconductor with a 2 eV bandgap. Spin-polarized calculations show that the system is antiferromagnetic in its ground state. The WO_3_ material is a semiconductor, as there is a bandgap of 2.5 eV between the valence and conduction bands. The Gd-doped WO_3_’s band structure shows few states across the Fermi level, which means that the material is metal or semimetal. After the doping of Gd, WO_3_ becomes the degenerate semiconductor with a bandgap of 2 eV. The energy difference between ferromagnetic (FM) and antiferromagnetic (AFM) configurations is negative, so the Gd-doped WO_3_ system is AFM. The pure WO_3_ is nonmagnetic, where the magnetic moment in the system after doping Gd is 9.5599575 μB.

## 1. Introduction

The exclusive ability to induce bistable electrical and optical characteristics in WO_3_ with different excitation sources makes it very promising among significant technological devices [1,2,3,4]. The element that has three oxygen atoms is called the perovskite-like structure. WO_3_ has useful applications in optical and spintronic devices [5,6,7]. It is used for the construction of semiconductor-based gas sensors (SGS) and electrochromic devices such as high-temperature superconductors (HTS), smart windows, solar cells, and water-splitting applications. The addition of electrons/holes could alter the characteristics of WO_3_. Additionally, the possibility of ion intercalation/deintercalation arises in several possible applications in rechargeable batteries. Especially, the photocatalytic activities and SGS properties of WO_3_ can be modified by doping transitional elements such as Au, Pd, and Pt. Rare earth and transition metal-doped WO_3_ systems show interesting magnetic properties that usually do not exist in undoped WO_3_ [8,9,10]. In this study, the magnetic, electronic, and optical properties of Gd-doped WO_3_ are investigated by using first principle calculations. Here is a brief description of previous works related to WO_3_. The monoclinic WO_3_ is the most communal and stable phase of WO_3_ with space group P21/n. The unit cell comprises 8W atoms and 24O atoms and holds 8O atoms at the corner in somewhat distorted cubic arrangements [11,12].

For the RT monoclinic, the direct bandgap calculated by using generalized gradient approximation (GGA) was increased initially as the volume decreased but, after that, decreases again with a further decrease in the volume. The cubic structure space groups Pm-3m contain basic structural characteristics but ignore distortion. The indirect bandgap of the cubic is smaller than the bandgap of the RT monoclinic. Low-temperature (LT) monoclinic structures with space groups Pc are other distorted forms of WO_3_. The unit cell of LT monoclinic contains 4W atoms and 12O atoms. The bandgap at a low-temperature (LT) monoclinic is, to some extent, greater than the RT monoclinic. This shows a direct bandgap with the conduction band minimum (CBM) and valence band maximum (VBM). The triclinic structures have space groups P-1 and contain 8W and 24O atoms. The bandgap of the triclinic is a direct bandgap. The orthorhombic structure of WO_3_ with a space group of P_mnb_ also has distorted oxygen-octahedrons [13,14]. The unit cell consists of 8W and 24O atoms. The bandgap is larger than the cubic and tetragonal ones but smaller than the monoclinic and triclinic structures [15].

The current studies showed that the assimilation of Gd^3+^ ion and other rare earth element ions in large bandgap semiconductor fallouts in boosting ferromagnetic properties inspired scientists on the way to rare earth elemental ion doping in various oxide nanomaterials for spintronics applications. Especially, the Gd^3+^ ion has more potential due to its optical and magnetic properties [16]. It was reported that WO_3_ is translucent in visible light, but strong absorption arises in near-infrared regions because of the phonon–electron interaction [17]. The reflectance and transmittance of WO_3_ material were noted in the range 400–2600 nm at P_tot_ = 10 and 30 mTorr. The dielectric function of the monoclinic and triclinic are comparable and cannot be separated by their dispersion relation. The optical gap is 2.5 to 2.6 eV, which is less than that of TiO_2_, and absorbs adequate visible radiations to produce a photocurrent. According to UV–Vis diffuse reflectance, WO_3,_ bare light absorbs at a wavelength of less than 460 nm, which gives an energy bandgap of 2.6 eV. In Gd-WO_3_, 4% absorption significantly transfers towards a longer wavelength from 460 to 470 nm along a bandgap of 2.64 eV. This perception is associated with the reality that hybridization occurs between O 2*p* and Gd 4*f*/5*d* orbitals alternatively and is incorporated into the WO_3_ lattice [18].

Bullet, Stashans, and Lunnel observed the effect of the interaction of alkali ions on the cubic, room temperature monoclinic and Perovskite structure of WO_3_ [19]. The WO_3_ electronic structure and sub-stoichiometry are informally connected to the properties of the structure. O 2*p* orbitals are present in the valence band, and W 5*d* orbitals are present in the conduction band. The phase transition outcome in the W 5*d* states causes changes in the energy gap. They concluded that the upward shift in the W 5*d* states changes the ideal cubic structure to a monoclinic structure and increases E_g_ from 1.5 to 2.45 eV [20].

The bandgap value of WO_3_ changes experimentally from 2.6 to 3.2 eV. This is due to the changes in the structure of WO_3_ [21]. The significance of the surface area and the interphase boundary is of great importance [22,23,24,25,26,27]. The insufficient oxygen WO_3-x_ is connected to the effect on the electrical properties and color, and the color changes from greenish to yellowish in WO_3_. In nanostructure WO_3_ films processed by reactive sputtering, E_g_ has linked to the O_2_ sputtering pressure and the vacancy of the O concentration. For the various phases of WO_3_ alternations in E_g_ with *d* orbital occupancy, the points of the VBM and CBM have been recognized. The calculation of cubic WO_3_ with the density functional theory (DFT) computations underreported at about 0.6 eV correlated with the value of the experimental value of 2.6 eV. In the dispersion of the band close to the region Nd–gap, the γ-WO_3_, δ-WO_3_, β-WO_3_, and ε-WO_3_ phases are observed, which are less by the initial calculations, which is due to the small difference in the lattice constant and short difference in the bond angle [17,28].

Even though only insufficient theoretical/experimental research has been done to discover the electronic/magnetic properties of RT monoclinic WO_3_, it is needed to explore the bandgap, energy band, and electron/hole reshuffle. Momentum density studies of WO_3_ have not been conducted up to now. WO_3_ exists in more than one crystalline form. The most common structure of WO_3_ is cubic, as for ReO_3_ [29,30,31]. The crystal structure of WO_3_ depends on the temperature when the temperature rises above 770 °C, its crystal shape is tetragonal; between 330 °C and 740 °C, it is orthorhombic; between 17 °C and 33 °C, it is monoclinic; and between −50 °C and 17 °C, it is triclinic [32,33,34]. Momentum density studies of WO_3_ have been not conducted to date. Even though only insufficient theoretical/experimental research has been done to discover the electronic/magnetic properties of RT monoclinic WO_3_, it is needed to explore bandgap, energy band, and electron/hole reshuffle. In the present work, the electronic, magnetic, and optical properties of Gd-doped WO_3_ are investigated by using first principle calculations. GGA+U approximation is applied to the orthorhombic structure to calculate the density of the states and optical and magnetic properties of WO_3_.

## 2. Simulation and Calculations

**Figure 1:**
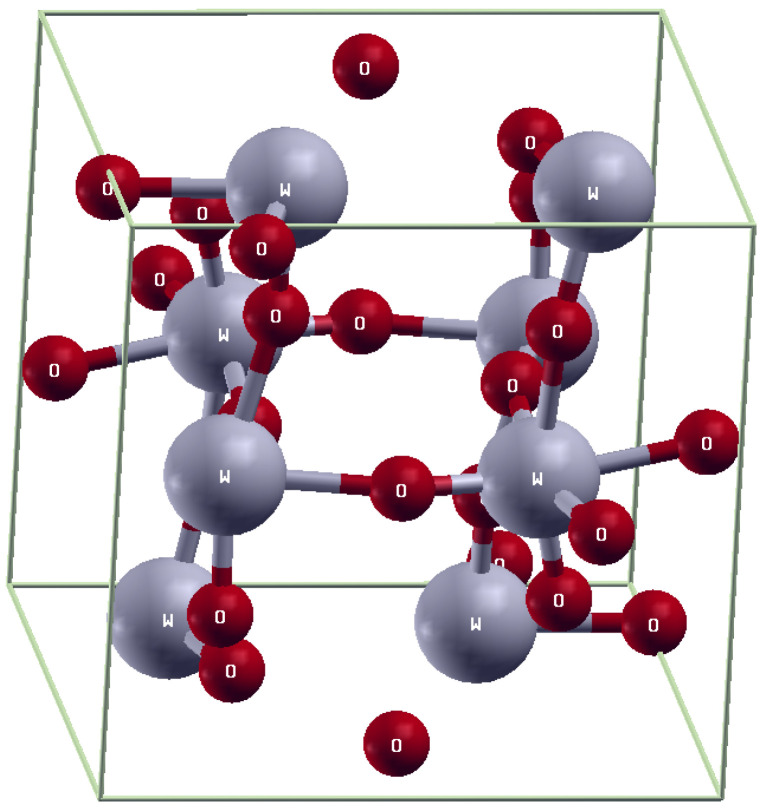
Orthorhombic structure of WO_3_.

The full potential linear augmented plane wave (FPLAPW) method with the WEIN2k code is used to calculate the optical, electrical, and magnetic properties of pure WO_3_ and Gd-doped WO_3_ [35]. The lattice parameters of pure WO_3_ are: a = 7.303, b = 7.5389, and c = 7.6896 A, and those of Gd-doped WO_3_ are a = 7.303, b = 7.5389, and c = 15.3724 A. The space group number of WO_3_ is 14P21/n, and K-mesh points 21 × 21 × 10 were used. The electronic charge density “ECD” expanded up to G_max_ = 12. In the interstitial region “IR”, the plane wave “PW” cut-off value is K_max_ = 6.5/RMT. The size of the supercell is 21 × 21 × 10, and it contains 16 atoms. The optical and magnetic properties of the orthorhombic-like structure WO_3_ are calculated using GGA+U approximation. The element that has three oxygen atoms is called the perovskite-like structure. The orthorhombic structure is shown in Figure 1.

## 3. Results and Discussion

**Figure 2:**
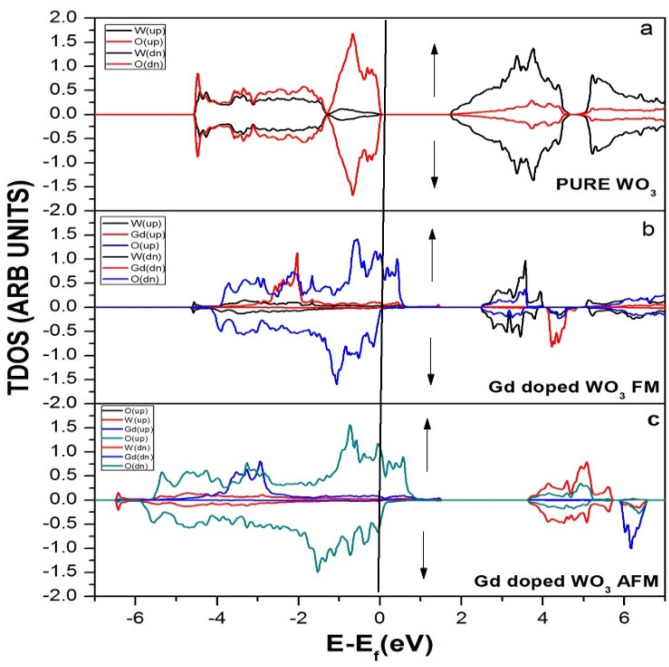
Density of the states (DOS) of (**a**) pure WO_3_, (**b**) Gd-doped WO_3_ FM, and (**c**) Gd-doped WO_3_ AFM by using GGA+U approximation.

Figure 2 represents the total density of the states of WO_3_ before and after the doping of Gd, a rare earth metal. There is no absolute energy taken along the x-axis but the Fermi energy. Fermi energy is an approach in quantum mechanics that usually mentions the highest filled states of single particles in a quantum system of noninteracting fermions at absolute zero temperature. In Figure 2a, no states reside in the Fermi level for pure WO_3_. In Figure 2b, after the doping of Gd, some states reside in the Fermi level when the system is ferromagnetic (FM), which means that all the unpaired electrons have the same spin directions. In Figure 2c, some states reside in the Fermi level after the doping of Gd when the system has antiferromagnetic materials (AFM), which means that all the unpaired electrons have a spin moment in the antiparallel direction.

**Figure 3:**
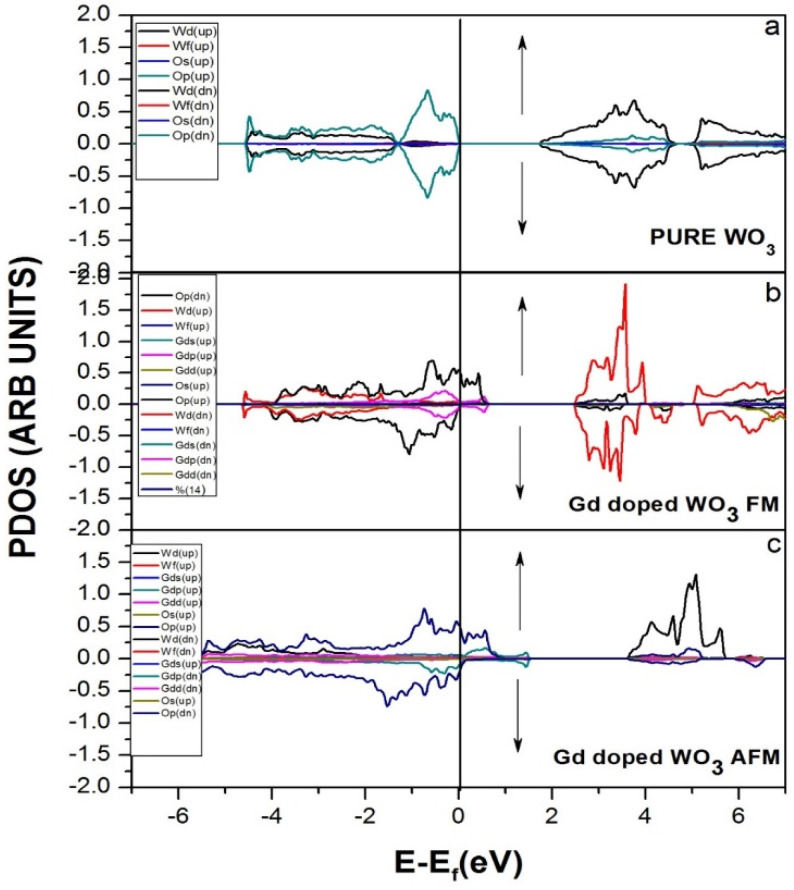
Projected DOS (PDOS) of (**a**) pure WO_3_, (**b**) Gd-doped WO_3_ FM, and (**c**) Gd-doped WO_3_ AFM by using GGA+U approximation.

**Figure 4:**
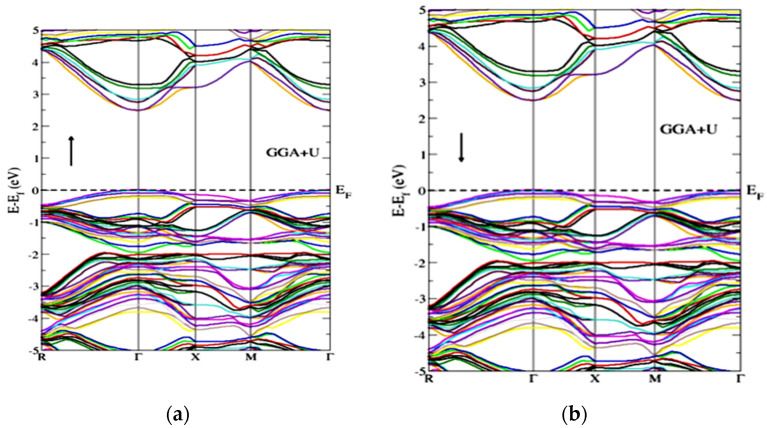
Band structure of pure WO_3_ for the (**a**) spin-up and (**b**) spin-down directions by using GGA+U approximation.

Figure 3 represents the partial density of the states. GGA+U approximation is used for the calculation of Gd-doped WO_3_. For calculating the partial density of the states, W has the states of *s*, *p*, *d*, and *f*, and O has *s* and *p*, while Gd has the *s*, *p*, *d*, and *f* states. Many clear states reside in the bandgap after the doping of the Gd rare earth metal. In the case of pure WO_3_, no states exist in the bandgap, as shown in Figure 3a, but after the doping of Gd, some states reside that cross the Fermi level, as shown in Figure 3b,c. Both calculations are done by using the GGA+U approximations. According to Figure 4, the material is a semiconductor, as there is a bandgap of 2.5 eV between the valence band and conduction band [36]. Figure 4 represents the band structure of pure WO_3_, which shows the direct bandgap. Figure 4a is the spin-up band, and Figure 4b is the spin-down direction.

**Figure 5:**
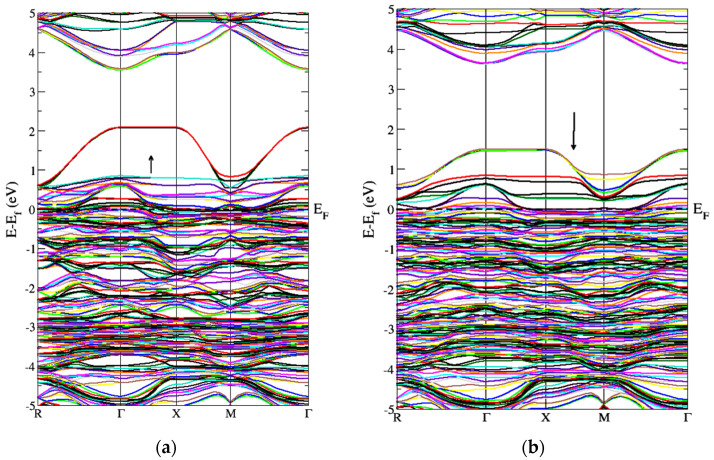
Band structure of Gd-doped WO_3_ for FM calculations (**a**) spin-up and (**b**) spin-down.

Figure 5 represents the band structure of Gd-doped WO_3_ for spin-up and spin-down in the FM configuration. It shows that few states cross the Fermi level, which means that the material is metal or semimetal. Figure 5 represents the gap inside the conduction band. From Figure 5, after the doping of Gd, WO_3_ becomes the degenerate semiconductor. The degenerate semiconductor shows a metallic character after high doping.

**Figure 6:**
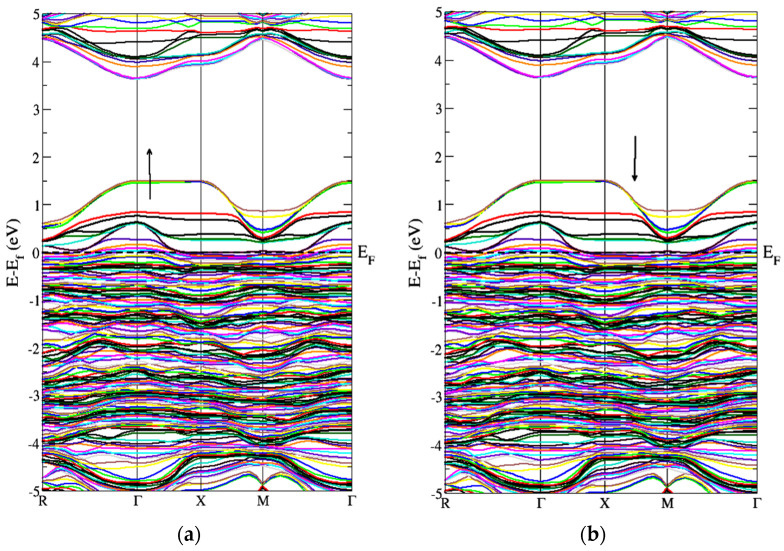
Band structure of Gd-doped WO_3_ for AFM calculations (**a**) spin-up and (**b**) spin-down.

Figure 6 represents the band structure of the Gd-doped WO_3_ by AFM calculations. Figure 6 is for the spin-up and spin-down directions after WO_3_ doping by Gd. As the states lie inside the Fermi level, it shows a metallic character after the doping of the Gd metal. Figure 6 also shows that, after the doping of Gd, the WO_3_ semiconductor becomes a degenerate semiconductor. As the energy difference between the FM and AFM configurations is negative, the Gd-doped WO_3_ system is AFM, according to the calculations, and the energy is calculated in millielectron volts. The pure WO_3_ is nonmagnetic, where the magnetic moment in the system after doping Gd is 9.5599575 μB (Table 1).

**Figure 7:**
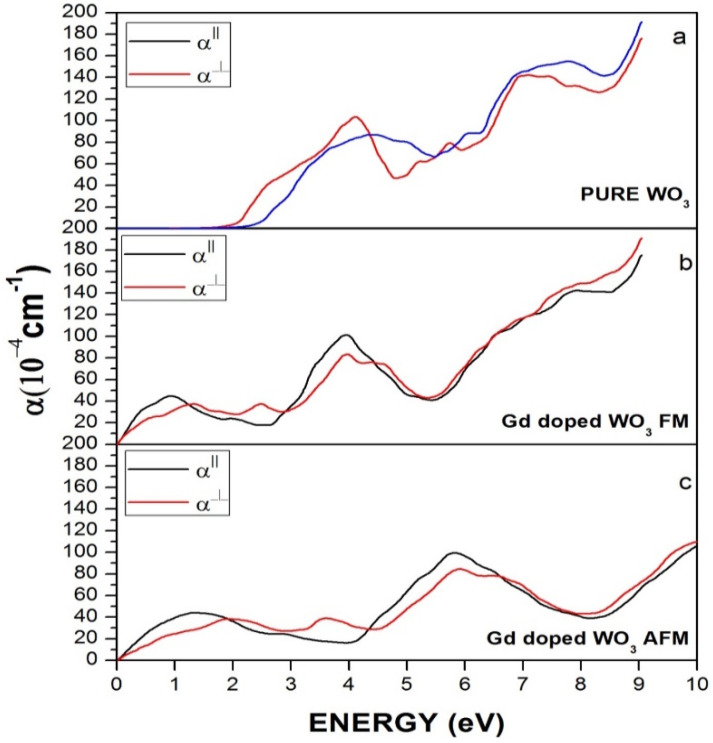
Absorption of (**a**) pure WO_3_, (**b**) Gd-doped WO_3_ for FM, and (**c**) Gd-doped WO_3_ for the AFM configuration.

Figure 7 represents the absorption coefficient of WO_3_ and Gd-doped WO_3_. Figure 7a indicates that, in pure WO_3_, the absorption increases gradually at energy 2 eV, which is the threshold energy for absorption and is equivalent to the bandgap energy. However, after the doping of the Gd rare earth element, there is a clear difference in the absorption of WO_3_, as the curve starts rising from 0 eV, as shown in Figure 7b,c, which means that the material becomes metallic or semi-metallic.

**Figure 8:**
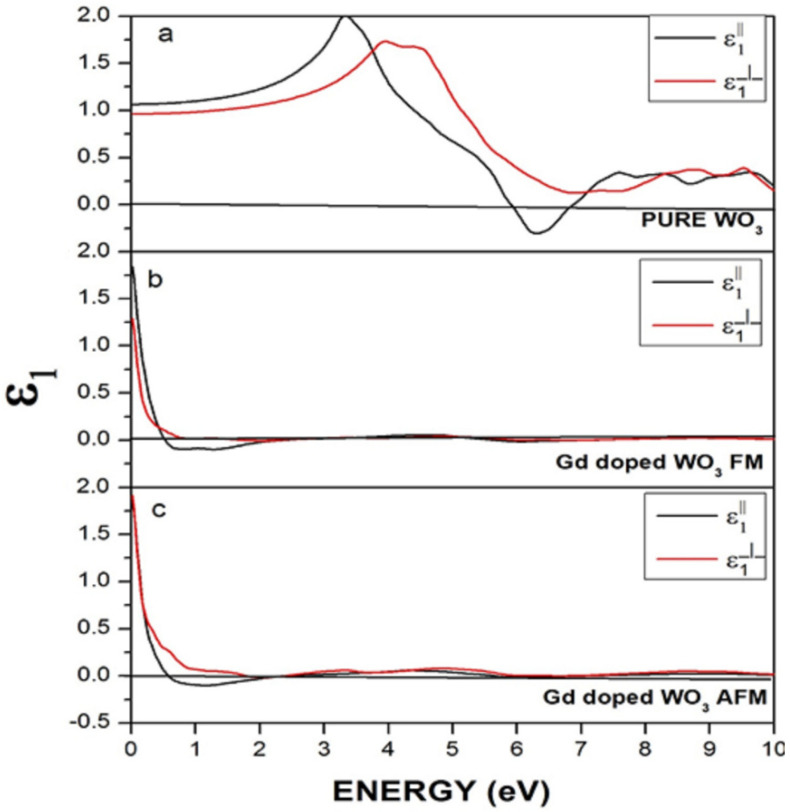
Real dielectric function of (**a**) pure WO_3_, (**b**) Gd-doped WO_3_ for FM, and (**c**) Gd-doped WO_3_ for the AFM configuration.

Figure 8 represents the real part of the dielectric function for pure and doped WO_3_ in FM and AFM configurations. In Figure 8a, the perpendicular component lies in the negative region in the energy range 6–7 eV, which shows that the material behaves reflective in this energy range and is transparent in the rest of the energy region. However, after the doping of Gd, as shown in Figure 7b,c, the states mostly lie above zero points, and very few states are below zero points. A clear reduction in the states below zero points happened. Figure 8b is FM Gd-doped WO_3_, and Figure 8c is Gd-doped WO_3_ in the AFM configuration. Gd causes a reduction of the states below zero points, which shows that the material became nonreflective.

**Figure 9:**
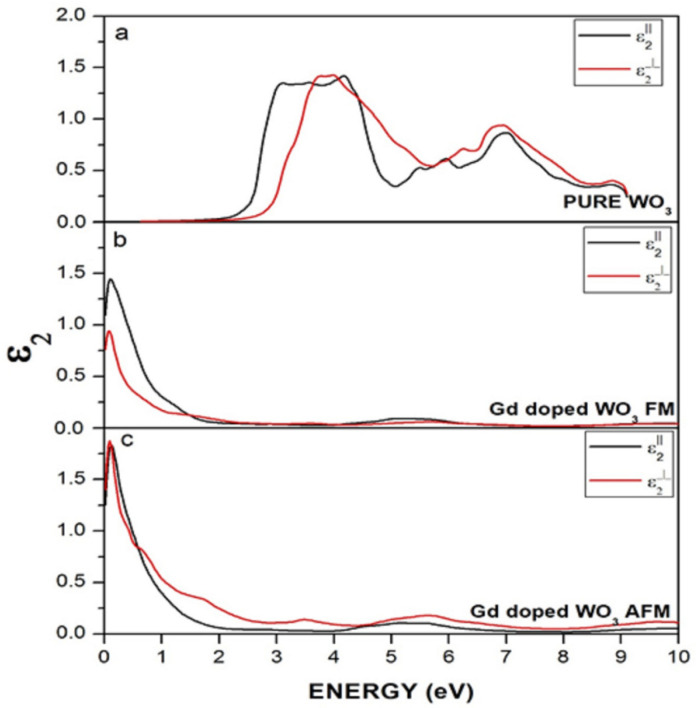
Imaginary dielectric function of (**a**) WO_3_, (**b**) Gd-doped WO_3_ for FM, and (**c**) Gd-doped WO_3_ for the AFM configuration.

A material’s complex dielectric function consists of two parts: real ε_1_(ω) and imaginary ε_2_(ω) (Figure 9). The absorption of photons is represented by the imaginary part of the dielectric function and electronic transition from valence towards the conduction band. The equation for ε_2_(ω) can be written as:

(1) $$\varepsilon_{2}\left(\omega \right)=\frac{4\pi^{2}e^{2}}{m^{2}\omega^{2}}\sigma_{\mathrm{ij}}\int \langle {i|M|j\rangle }^{2}f_{i}\left(1-f_{i}\right)\delta \left(E_{j}-E_{i}-\omega \right)d^{3}k$$

where M represents the dipole matrix, the free electron is represented by m, for the initial and final states, the i and j symbols are used, the ith state Fermi distribution is represented by f_i_, and E_i_ and E_j_ are the energy of the free electrons in the initial and final states.

## 4. Conclusions

In the present work, the electronic, magnetic, and optical properties of pure WO_3_ and Gd-doped WO_3_ were calculated by using the GGA+U approximation for exchange–correlation energy. The energy difference between the FM and AFM configurations showed that the Gd-doped WO_3_ system is AFM. Pure WO_3_ is a nonmagnetic semiconductor with a bandgap of 2.5 eV. The system becomes a degenerate semiconductor after the doping of the rare earth element Gd in WO_3_. In the real dielectric function of WO_3_, the perpendicular component lies in the negative region in the energy range 6–7 eV, which shows that the material behaves as reflective in this energy range and is transparent in the rest of the energy region. The spin-polarized calculations showed that the system is antiferromagnetic in its grounded state. The WO_3_ material is a semiconductor, as there is a bandgap of 2.5 eV between the VB and CB. Pure WO_3_ is nonmagnetic, where the magnetic moment in the system after doping Gd is 9.5599575 μB.

## Figures and Tables

**Table 1:** Magnetic properties of Gd-doped WO_3_.

Compound	Supercell Size	△E = EAFM − EFM (meV)	Coupling	$\mathrm{Spin}\;\mathrm{Magnetic}\;\mathrm{Moment}\;\mathrm{in}\;\mathrm{Supercell}\;(\mu_{B})$
Gd: WO3	21×21×10	−1.05815002	AFM	9.5599575

## Data Availability

The datasets generated during and/or analyzed during the current study are available from the corresponding author upon reasonable request.
